# Ultrasound and CT Features of Isolated Gallbladder Torsion With a Suspected Congenital Anomaly in a Cat: An Imaging Diagnosis

**DOI:** 10.1111/vru.70267

**Published:** 2026-11-14

**Authors:** Hana Moserová, Radka Dvořáková, Florian Lein, Michaela Junášková, Jana Lorenzová, Pavel Proks

**Affiliations:** ^1^ Department of Diagnostic Imaging Small Animal Clinic Faculty of Veterinary Medicine University of Veterinary Sciences Brno Brno Czech Republic; ^2^ Clinical Department for Small Animals and Horses, Diagnostic Imaging University Clinic for Small Animals, University of Veterinary Medicine Vienna Vienna Austria; ^3^ Department of Internal Medicine Small Animal Clinic Faculty of Veterinary Medicine University of Veterinary Sciences Brno Brno Czech Republic; ^4^ Department of Surgery and Orthopedics Small Animal Clinic Faculty of Veterinary Medicine University of Veterinary Sciences Brno Brno Czech Republic

**Keywords:** bile duct, computed tomography, gallbladder, torsion, ultrasonography

## Abstract

An 8‐month‐old domestic short‐haired cat was presented to the emergency room with an episode of peracute vomiting and abdominal pain. Ultrasound and computed tomography (CT) examination revealed an abnormally positioned and distended gallbladder with a thickened gallbladder wall. A presumptive diagnosis of gallbladder torsion was made and subsequently confirmed during laparotomy. Although uncommon, gallbladder torsion should be considered a differential diagnosis in feline patients with clinical signs of abdominal pain and peracute vomiting.

## 1 Signalment, History, and Clinical Findings

An 8‐month‐old intact female domestic short‐haired cat, with a body weight of 2.5 kg, was presented to the emergency room with peracute repeated vomiting lasting the past 12 hours and a painful abdomen. The cat had no history of previous diseases, was kept indoors, and was appropriately vaccinated and dewormed.

Clinical examination revealed tachycardia (280 beats per minute), tachypnea (40 breaths per minute), and rectal temperature of 38°C. Mucous membranes were pink, and auscultation was unremarkable. The patient was very painful and vocalizing during abdominal palpation, markedly during the palpation of the cranial abdomen. Complete blood count and serum biochemistry (Procyte DX and Catalyst One; IDEXX) were within the reference intervals. The feline leukemia virus and feline immunodeficiency virus test were negative.

## 2 Imaging, Diagnosis, and Outcome

Ultrasound examination of the abdominal cavity was performed using a Canon Aplio i700 (Canon Medical Systems Corporation, Tochigi, Japan) ultrasound machine with microconvex (4–11 MHz) and linear (8–22 MHz) transducers. A small amount of free anechoic fluid was detected predominantly in the cranial abdomen around the gallbladder. The gallbladder was not situated in its expected anatomical location; instead, it was positioned caudally to the caudal margins of the hepatic lobes, between the liver and the stomach wall (Figure 1A). The gallbladder was also markedly filled with anechoic content with occasional echogenic debris, with maximal dimensions of 32 × 16 × 16.5 mm, and the gallbladder wall was significantly thickened (up to 4.3 mm) with visible stratification and visible constriction in the area of the anatomical location of the neck (Figures 1B and 2A). Color Doppler and SMI Doppler evaluation of the gallbladder wall did not show any intramural blood flow (Figure 2B). The fat surrounding the gallbladder was significantly hyperechoic and edematous. On the basis of these findings, gallbladder torsion was suspected, with differential diagnoses like gallbladder infarction or necrosis considered less likely. Additional abdominal imaging and subsequent surgery in case of diagnosis confirmation were recommended.

**FIGURE 1:**
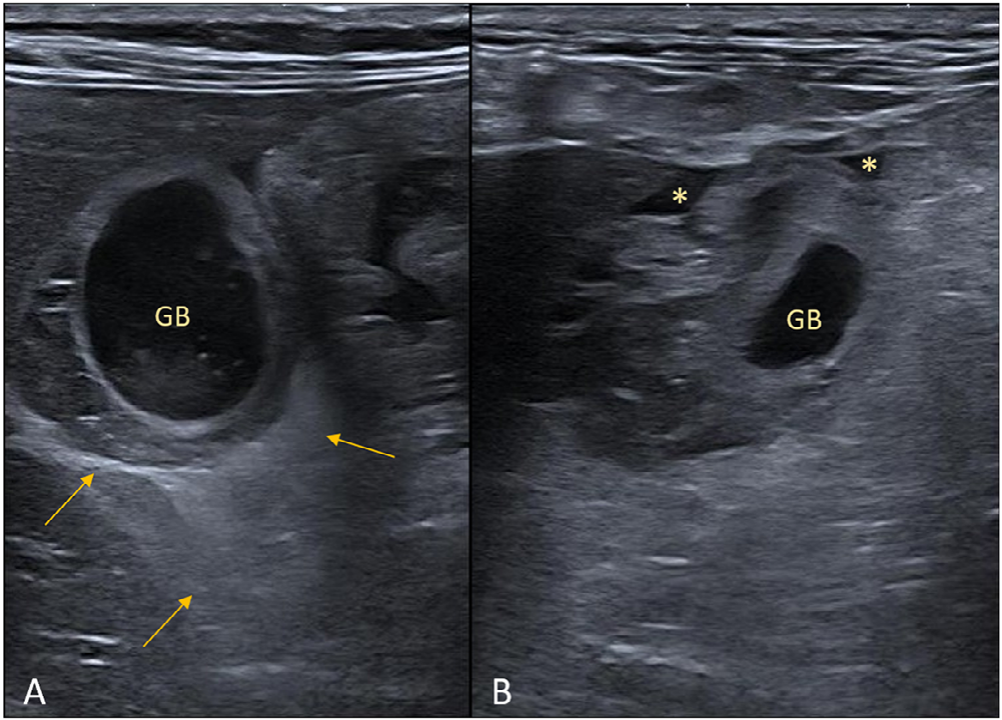
Ultrasound examination of the right cranial abdomen in longitudinal plane, linear transducer (8–22 MHz). (A) The gallbladder (GB) is identified between the caudal liver lobe margin and stomach, with marked wall thickening and hyperechogenicity of the surrounding fat (arrows). (B) The gallbladder at the level of the gallbladder neck, manifesting a severely thickened wall, surrounded by small amounts of anechoic free fluid (asterisks).

**FIGURE 2:**
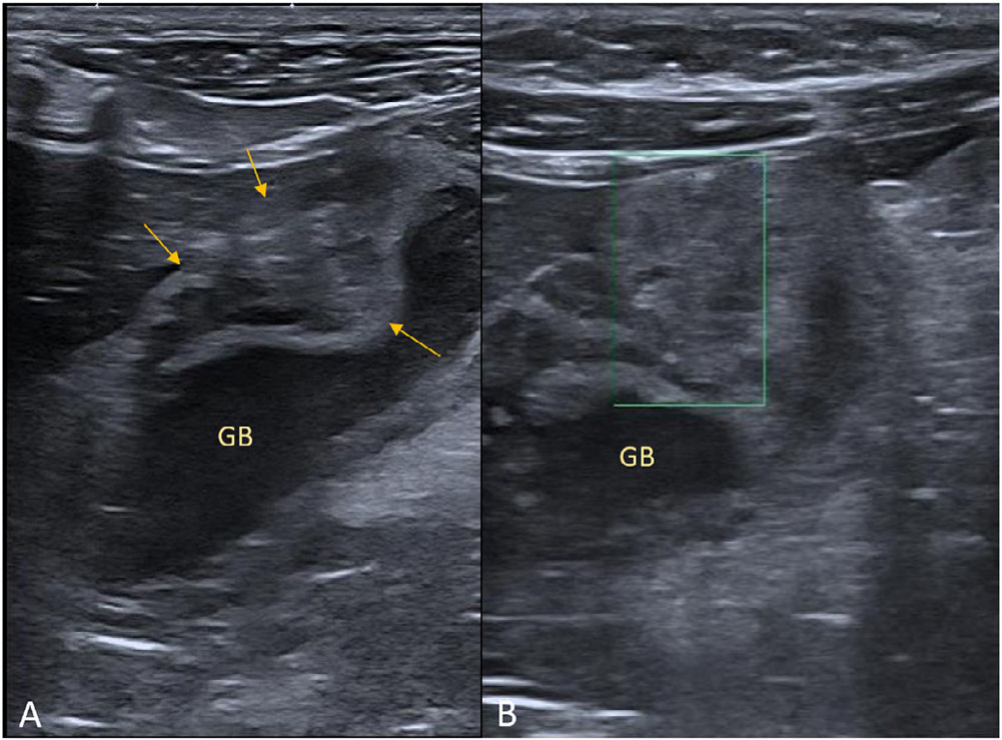
Ultrasound examination of the right cranial abdomen in oblique transverse plane, linear transducer (8–22 MHz). (A) Full‐length view of the gallbladder (GB), with markedly thickened wall most prominently in the area of the neck (arrows). (B) SMI Doppler examination of the thickened gallbladder wall, with no detection of flow in the gallbladder wall.

A computed tomography (CT) examination of the abdomen was performed using an 80‐slice scanner (Aquilion Lightning, Canon Medical Systems Corporation, Tochigi, Japan). Pre‐ and post‐contrast 0.5 mm images were acquired and reformatted using low (soft tissue) and high (bone) frequency algorithms. A 5 mL bolus of intravenous iodinated contrast agent (Xenetix 350 mgI/mL, Guerbet, France) was manually administered to acquire post‐contrast images, with post‐contrast scans being performed in a nonspecific angiographic phase (immediately after finishing the administration) and late phase (120 s after administration).

The gallbladder fundus was localized caudally from the caudal edges of the liver. The gallbladder was triangular in shape, with the neck detected at the level of the caudal liver lobe margins (Figure 3A,C). Additionally, a hypodense tubular structure was detected (Figure 3A–C), continuing from the gallbladder neck dorsally through the area of normal anatomical location of the gallbladder (between the right medial lobe and the quadratus lobe), and connecting to the normal anatomical course of the cystic duct and common bile duct coursing ventrally from the portal vein. No visually appreciable contrast enhancement of the gallbladder wall area was detected on post‐contrast scans. Around the gallbladder, discrete linear densities between fat folds were present (“fat stranding” sign). The rest of the abdominal cavity was unremarkable.

**FIGURE 3:**
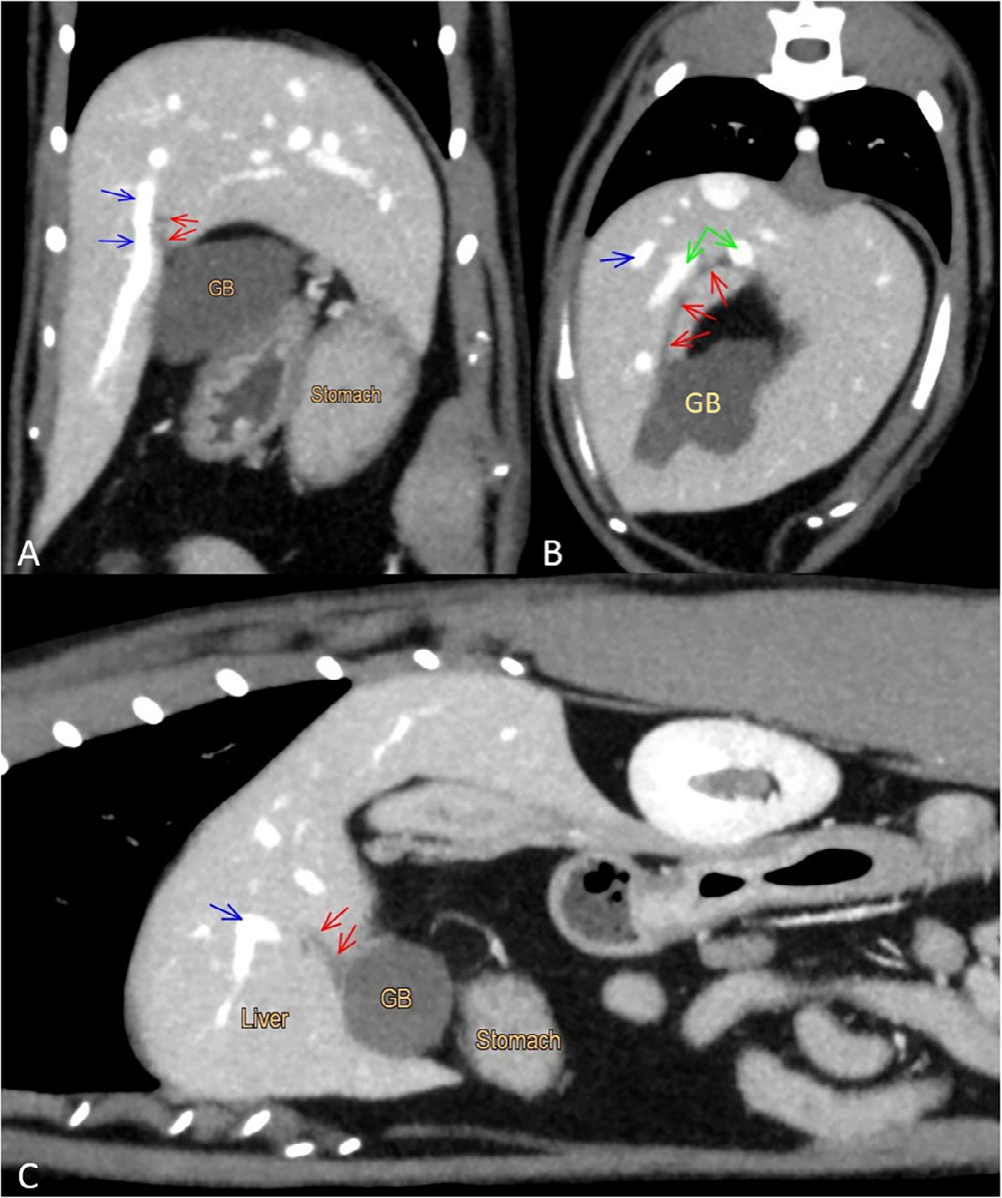
Dorsal (A), oblique transverse (B), and sagittal (C) CT images of the abdomen, post contrast scan in nonspecific angiographic phase, soft tissue algorithm. The gallbladder (GB) is positioned caudally to the caudoventral liver margins, with no detection of contrast enhancement. A long hypodense tubular structure is visualized connecting the gallbladder neck with the area of normal anatomical location of the gallbladder and cystic duct (red arrows). The left and right branch of the portal vein is marked with green arrows; hepatic veins are marked with blue arrows.

On the basis of the findings described above, the diagnosis of gallbladder torsion, possibly associated with abnormal gallbladder attachment to the liver, was suspected. The patient was referred to the Department of Surgery and Orthopedics for an emergency laparotomy. A video file of the second post‐contrast phase in transverse view is available in Video S1.

Preoperatively, ampicillin–sulbactam (ampicillin–sulbactam inj. 10 × 1500 mg, 20 mg/kg) and etamsylate (Hemosilate 125 mg/mL, 12.5 mg/kg) were administered. The cat was premedicated with fentanyl (Fentanyl Kalceks 0.05 mg/mL inj. sol., 1 µg/kg), and anesthesia was induced with propofol (Proposure 10 mg/mL, 4 mg/kg) and maintained with isoflurane following standard protocol. After the standard patient preparation, craniomedian laparotomy in the linea alba was performed. The gallbladder was rotated approximately 360° clockwise in its long axis, with the torsion centered on the area of the gallbladder neck and cystic duct (Figure 4A). Subsequent vascular constriction and incipient necrosis of the gallbladder wall were noted. The surface of the gallbladder was not attached to the liver lobes or elongated mesentery. Furthermore, a markedly elongated tubular structure was detected (Figure 4B) in a location corresponding to the previous CT finding. The visualized segment of this structure, unattached to the liver, together with the gallbladder, was resected and sent for histopathological examination. Ligation of the tubular structure was performed using polyfilament suture material (Vicryl 3‐0) before its resection, and the stump was closed with cruciate suture using nonabsorbable monofilament suture material (Prolene 3‐0). After closure and dressing of the surgical wound, the patient was transferred to the ICU, where infusion, antiemetic, and analgesic therapy was provided, with an administration of maropitant (Cerenia 10 mg/mL, 1 mg/kg) and buprenorphine (Bupaq Multidose 0.3 mg/mL inj. sol., 10 µg/kg).

**FIGURE 4:**
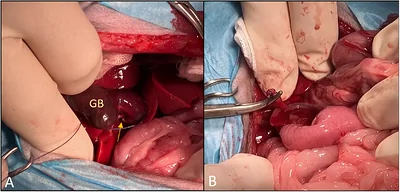
Intraoperative images of the gallbladder (A) and tubular structure connected to the gallbladder neck (B). (A) The gallbladder (GB) is of a triangular shape, with incipient wall necrosis; the location of the torsion at the level of the neck is marked with an arrow. (B) The elongated tubular structure connecting the gallbladder neck with the area of normal anatomical location of the common bile duct is captured in the Pean forceps.

Histologic examination revealed almost complete destruction of the wall with massive congestion and numerous foci of extravasation, groups of chronic inflammatory cellularization, and areas of fibrinoid necrosis. These findings were consistent with a diagnosis of a circulatory defect—torsion. No signs of dysplasia or malignancy were detected in the scope of the supplied material.

Postoperative recovery was uneventful. The cat resumed voluntary food intake a few hours after surgery and was discharged to home care the next day. The patient appeared clinically healthy on follow‐up examination 10 days after surgery. The follow‐up abdominal ultrasound revealed persisting mild signs of inflammation in the right cranial abdomen (fat hyperechogenicity, minimal amount of free fluid). No distention of the common bile duct or intrahepatic bile ducts was noted.

## 3 Discussion

Gallbladder torsion is a rare, acute, and life‐threatening condition in humans and animals. In dogs and cats, the gallbladder is physiologically located in the *fossa vesicae felleae* between the quadrate lobe and the right medial hepatic lobe, to which it is attached by connective tissue [1, 2, 3]. Gallbladder torsion may occur in association with torsion of the hepatic lobes; in these cases, the connective tissue attachment to the *fossa vesicae felleae* is preserved. Isolated gallbladder torsion typically occurs in patients with a congenital anatomical abnormality called a floating/wandering gallbladder [4, 5]. Although the floating gallbladder is attached by an elongated mesentery, the wandering gallbladder lacks a fibrous attachment to the liver or a connection to the mesentery, with the only fixation being the cystic duct and vasculature [5, 6].

Isolated gallbladder torsion is a rare condition in small animals; only four cases in canine patients and one case in a feline patient have been previously described in the reviewed literature [6, 7, 8, 9, 10]. The reported predisposing factor, the anatomical abnormality of the wandering/floating gallbladder, has been observed in all published canine cases. In contrast, there was no evidence of this anatomical abnormality in the published feline case of isolated gallbladder torsion, suggesting a different etiology. Moreover, the torsion was centered on the gallbladder neck and cystic duct in canine cases with a wandering/floating gallbladder present, while being centered on the gallbladder fundus in the feline case, with a described connection of the gallbladder to the omentum [10].

All previously documented patients presented with an acute onset of vomiting and severely painful cranial abdomen. Although these findings are nonspecific, gallbladder torsion should be considered in the differential diagnosis, especially in young animals [7, 9]. The bloodwork in documented canine cases was mostly within normal limits or with mild changes, such as mild elevation of alanine aminotransferase [8]. Alternatively, more pronounced changes in serum biochemistry were reported in the feline case of isolated torsion [10].

With the bloodwork within normal limits, together with ultrasound and CT findings, namely, the abnormal position of the gallbladder, severe wall thickening, and a lack of fibrous attachment of the gallbladder wall, our case has more resemblance to the described canine cases of isolated torsion than to the feline one. The authors suggest that this might reflect a similar etiology in our case, namely, the absence of attachment of the gallbladder to the right medial hepatic lobe or mesentery, indicative of a form of wandering gallbladder. The similarity with the reported canine cases further relates to age, as most of the patients were presented at a young age of 11 months [7, 8, 9]. This also corresponds with information from human medicine, where children are the most predisposed group [11].

Histopathology of the gallbladder revealed signs of chronic inflammation, similar to the previously described case of isolated torsion in a cat [10]. Although these findings could represent background chronic changes, an inflammatory process preceding and associated with the torsion cannot be ruled out. The finding of a tubular structure in the area of the normal anatomical location of the gallbladder, connecting the gallbladder neck with the common bile duct, could represent a stretched or elongated cystic duct and common bile duct. Alternatively, this structure was also hypothesized to be an anomalous form of prolonged cystic duct/bile duct based on macroscopic morphology, with a possible contribution to increased gallbladder mobility. However, histologic examination of this structure was not disclosed. Gallbladder and bile duct malformations are anecdotally common in cats, with two cases of anomalous extrahepatic bile ducts without associated clinical manifestations being reported recently [12].

An untreated gallbladder torsion can lead to gallbladder necrosis and rupture and the associated biliary peritonitis, or hemoabdomen, namely, in association with liver lobe torsion [13]. Early diagnosis and subsequent surgical management can prevent or limit the development of these life‐threatening complications. However, the presurgical diagnosis of this condition is not easy, as shown by data from human medicine, according to which a preoperative diagnosis was made in only 26% of patients [14]. A similar trend is currently observed in companion animal medicine, where only one of the four published canine cases provides a preoperative diagnosis [7], as well as our case. The remaining cases of isolated gallbladder torsion in dogs and cats were diagnosed on the basis of exploratory laparotomy. These statistics are likely due to both the lack of awareness of isolated gallbladder torsion as a cause of acute abdominal symptoms and the low number of published case reports presenting typical imaging findings [9].

In the previously described case reports, a combination of imaging methods was used to diagnose gallbladder torsion: X‐ray and ultrasonography [6, 8, 10], or ultrasonography and CT [7, 9]. The previously reported preoperative diagnosis was determined on the basis of ultrasonography and CT findings, similarly to our case [7]. Gallbladder distension, thickened wall, horizontal orientation, and abnormal positioning with the gallbladder neck positioned more cranially than the body are sonographic findings suggestive of an isolated torsion described in dogs [7, 9], and some of these findings were also present in our patient. An important differential diagnosis for these findings (apart from abnormal positioning) is acute cholecystitis [7]. Radiographic loss of detail in the cranial abdomen, as well as sonographic signs of peritonitis (hyperechogenic fat, small amounts of peritoneal effusion) in this location, are considered nonspecific, as a number of other diseases (acute hepatitis, pancreatitis, and erosive/ulcerative gastritis) may be associated with similar findings [7, 8, 9].

Additionally, color and SMI Doppler examination was performed in our patient, with no blood flow being visible in the area of the thickened gallbladder wall. Although the absence of vascularization in Doppler imaging has been described in human medicine as a possible sign of torsion [15], this examination has not yet been performed in previous case reports regarding companion animals. Doppler examination or contrast‐enhanced ultrasonography (CEUS) may represent possible methods helpful in differentiating this disease from other pathologies; however, a lack of blood flow on Doppler, SMI, or CEUS imaging could also indicate gallbladder infarction or necrosis of a different etiology (e.g., necrotizing cholecystitis) [16, 17]. Further studies are warranted to evaluate the clinical reliability and utility of these techniques in diagnosis of gallbladder torsion in small animals.

Advanced imaging methods (CT, magnetic resonance imaging) facilitate the diagnosis of this disease, as they allow for clearer visualization of the organ position and reportedly also for detection of twisting of the cystic artery with surrounding fat (whirl‐sign) or sudden narrowing of the cystic duct (beak‐sign) [7, 9, 14]. However, these signs were not observed in the presented case. Given the comparatively lower availability of these modalities, the higher cost, and the need for anesthesia, ultrasonography with the use of Doppler imaging seems to be a promising and advantageous imaging modality for this disease in veterinary medicine, according to the authors.

When treated early, gallbladder torsion has a good prognosis; in human medicine, a mortality rate of 6% is reported, mainly due to multiple comorbidities in elderly patients [14]. In the aforementioned study, no deaths were recorded in any of the patients with a preoperative diagnosis, and the course of the case presented here was consistent with this. On the contrary, bile leakage and the development of peritonitis are associated with a worse prognosis, highlighting the need for a prompt and accurate diagnosis and treatment [13].

## Author Contributions

**Hana Moserová**: writing – original draft, writing – review and editing, conceptualization, investigation. **Radka Dvořáková**: writing – review and editing, investigation, formal analysis. **Florian Lein**: writing – review and editing, formal analysis, conceptualization. **Michaela Junášková**: investigation, conceptualization. **Jana Lorenzová**: supervision, formal analysis. **Pavel Proks**: supervision, project administration, writing – review and editing.

## Disclosure

The authors have nothing to report.

## Ethics Statement

The animal usage guidelines were followed. An informed consent form was signed by pet owners, allowing the use of patient data upon admission. All procedures were performed as part of veterinary clinical care; ethical approval from a committee was therefore not required.

## Conflicts of Interest

The authors declare no conflicts of interest.

## Supporting information




**Supporting File**: vru70267‐sup‐001‐Vedio S1.webm

## Data Availability

The data supporting the findings in this manuscript are available from the corresponding author upon reasonable request.
